# Comparing the role of social connectivity with friends and family in depression among older adults in China: evaluating the moderating effect of urban–rural status

**DOI:** 10.3389/fpsyt.2023.1162982

**Published:** 2023-05-11

**Authors:** Yuxuan Gu, Shahmir H. Ali, Aimei Guo

**Affiliations:** ^1^Center for Gerontology Research, Department of Social Security, Nanjing Normal University, Nanjing, China; ^2^School of Global Public Health, New York University, New York, NY, United States

**Keywords:** social connectivity, friends, family, depressive symptoms, urban, rural

## Abstract

**Background:**

Social connectivity and support can reduce depressive symptoms. Few studies have examined urban–rural differences in the relationship between social support and depressive symptoms in the context of urbanization for Chinese older adults. The overall aim of this study is to examine urban–rural differences in the relationship between family support and social connectivity on depression among Chinese older adults.

**Methods:**

This cross-sectional study used data from the 2010 Sample Survey on Aged Population in Urban/Rural China (SSAPUR). Depressive symptoms were measured using the Geriatric Depression Scale short-form (GDS-15). Family support was measured by structural, instrumental, and emotional support. Social connectivity was measured using the Lubben Social Network Scale-6 (LSNS-6). Descriptive analysis was conducted using chi-square and independent *t*-tests to examine urban–rural differences. Adjusted multiple linear regressions were conducted to examine the moderating effect of urban–rural status on the association between types of family support and social connectivity with depressive symptoms.

**Results:**

In rural areas, respondents who felt their children exhibited filial piety (*β* = −1.512, *p* < 0.001) and had more social connectivity with family (*β* = −0.074, *p* < 0.001) were more likely to report fewer depression symptoms. In urban areas, respondents who received instrumental support from their children (*β* = −1.276, *p* < 0.01), who thought their children exhibited filial piety (*β* = −0.836, *p* < 0.01), and who had more social connectivity with friends (*β* = −0.040, *p* < 0.01) were more likely to report fewer depression symptoms. In the fully adjusted regression model, social connectivity with family was associated with decreased depressive symptoms, although to a lesser degree among urban-dwelling older adults (urban–rural interaction effect, *β* = 0.053, *p* < 0.05). Social connectivity with friends was similarly associated with decreased depressive symptoms, although this effect was greater among urban-dwelling older adults (urban–rural interaction effect, *β* = −0.053, *p* < 0.05).

**Conclusion:**

The results of this study suggested that older adults both in rural and urban areas with family support and social connectivity were associated with fewer depression symptoms. Differences observed in the role of family and friend social connectivity by urban–rural status may provide practical information for developing targeted social support strategies for improving depressive symptoms among Chinese adults, and call for further mixed-methods research to disentangle mechanisms behind these differing associations.

## 1. Introduction

Depression, one of the most common geriatric psychiatric disorders and a significant contributor to reduced physical and mental health, has an average expected global prevalence of 31.74% (1). Notably, the pooled prevalence of depression is higher among low-middle income countries compared with high-income countries (1). Depression is a leading cause of disability and mortality worldwide and is a major contributor to the overall global burden of disease, which was also confirmed in older adults (2–4). In China, the depression is expected to affect about 33% of the total older adult population (5), poses a high individual and health system burden in the country (6, 7). With China’s dramatically aging population, addressing the physical and mental health burden of older adults is of pressing concern.

Although there has been significant research into risk factors of depression, less is known on risk factors specific to the Chinese context, which is crucial to inform policy recommendations for the development of effective prevention efforts. Depression is often a result of a complex interaction of biological, psychological, and social factors including physical health (8, 9), genetic factors (10), stress (11), sleeping disorder (12), social isolation and loneliness (13), lack of exercise or physical activity (14–16), and functional limitations (17). In particular, poor physical health, poor social support, and bereavement have been observed as particularly strong contributors to depression incidence among older adults (18). However, among these complex contributing factors, social support and connectively is notably important given its modifiability through targeted social interventions among older adults.

Social isolation and loneliness are widespread issues, including in China (19), with some countries reporting that up to one in three older people feel lonely (9). Social connectivity is a crucial component of healthy living, however as one ages, this can often result in spending more time alone as a result of living alone, the loss of family or friends, etc. A large body of research shows that social isolation and loneliness have a serious impact on older people’s longevity, physical and mental health, and quality of life. Studies show that loneliness and social isolation are associated with higher rates of depression (20); improving social connectivity and family support can act as protective factors against social isolation (21, 22).

Research has been done on the association between family and social support and the health of older adults extensively. A review conducted in Asia found that good overall social support, having a spouse or partner, living with family, having a large social connectivity, having more contact with family and friends, having emotional and instrumental support, good support from family, and satisfaction with social support are associated with fewer depressive symptoms among community-dwelling older adults (23). With regards to the source of social support, support from family was found to lower depressive symptoms in three studies (24–26), while one study found that friends’ support is associated with fewer depressive symptoms (27). Compared with findings in Western countries (which have a comparatively greater emphasis on social support from spouses and friends among older adults with depression depression), immediate and extended family play a crucial role as social supports for Asian older adults (28). This difference can in part be explained by the values such as familism and collectivism that are central tenants to many Asian cultures, including in China (29). For example, filial piety is a prevalent system of values across Asia in which older adults tend to rely on children and family members for care in old age, thus acting as foundational sources of social support. At present, there are relatively few domestic studies on the influence of social support from friends on the mental health of the elderly. However, in countries such as China, urbanization and industrialization are resulting in dramatic shifts in how family members of older adults are able to interact with each other, including a decrease in multiple generational living, and greater logistical difficulties and less expectations for children to look after parents (30, 31). Social support dynamics may be changing in Asian countries in light of this industrialization, urbanization, shifting social norms. It has been established that there’s a higher rate of depression of older adults in rural areas than urban areas (32–37) and health status, social support and social participation accounted for some of the rural–urban difference (34). However, little is known on urban–rural differences in the relationship between social connectivity and depressive symptoms in the context of urbanization for Chinese older adults, which is crucial to inform social and health interventions that are tailored to the unique social dynamics in each setting.

The overall aim of this study is to examine the relationship between family support, social connectivity (with both family and friends) and depression among Chinese older adults, and whether living in an urban versus a rural environment may affect these associations. We hypothesized that greater family support and social connectivity are positively associated with fewer depression symptoms. Especially, we expect that social connectivity with friends will be particularly important in urban areas (given the potential for more dispersed family living and greater diversity in social networks), while social connectivity with family will be particularly important in rural areas (where multigenerational living in larger households may be more prominent).

## 2. Method and design

### 2.1. Study design and dataset

This study used a dataset derived from the 2010 Sample Survey on Aged Population in Urban/Rural China (SSAPUR) conducted by the China Research Center on Aging (CRCA). Details on the study methodology have been described elsewhere (38). Briefly, SSAPUR is the first nationwide survey of the population aged 60 or older covering 20 provinces randomly selected from a total of 31 provinces in mainland China sponsored by the Chinese government. The study employed a multistage sampling strategy using a probability proportional to size (PPS) approach in which four counties and four cities were randomly selected from each of sampled provinces according to the estimated aged population in 2010. Four townships in rural areas and four street offices in urban areas were then randomly selected from each sampled county or city. In total, 640 townships and street offices were selected, and three to four villages in each sampled township (or three to four residential committees in each sampled street office) were further selected. Ten households with at least one person aged 60 or older were finally selected and only one older person in the sampled household was interviewed in at-home visits by trained interviewers. The response rate was 99.9%—remarkably high because of government sponsorship of the study, particularly effective in building trust among older populations in China. The survey included 20,023 respondents aged 60 or older, of which 10,069 were from urban areas. For the urban sample, in addition to collecting data on demographics, socioeconomic conditions, family and social support, practice, and health conditions, the survey also included several questions on community care. The core dataset collected in Jiangsu Province was used to conduct data analysis in the current study. We selected data from Jiangsu province as our research sample. According to the data of the 7th national census, the resident elderly population in Jiangsu province aged 60 and above is 18,505,300, accounting for 21.84% of the total population, which is 3.14 percentage points higher than that of the whole country and the highest among the top four economic provinces in terms of GDP. And in general, the industrialization and urbanization process of Jiangsu is earlier than many other provinces, some cities in Jiangsu are more strictly enforced family planning, the birth rate has been low for a long time, and the aging of the Jiangsu region is among the highest in the country, which can provide a reference for the country. The inclusion criteria for the present study were participants who are ≥60 years of age and who responded to the items for depressive symptoms and items for family support and social connectivity in 2010. We exclude variables with missing values for key variables; the final sample in this study included 824 persons.

### 2.2. Measures

#### 2.2.1. Outcome variables: depression symptoms

Depressive symptoms were measured by using the Geriatric Depression Scale short-form (GDS-15). Among the various depression screening instruments, the GDS was the first developed for the aging population (39). The 15-item GDS-SF is derived from the 30-item GDS and is a popular depression screening tool for older adults (40). Respondents are asked to indicate whether they have experienced specific symptoms during the past week (e.g., drop activities and interests, feel that life is empty, get bored), reporting either “yes” or “no” and was coded as “1” and “0.” Higher scores reflect more severe mental health problems. A continuous added-up GDS score was used as an outcome variable in our analysis. The scale is found to carry satisfactory internal consistency for the study sample. The Cronbach’s alpha was 0.802.

#### 2.2.2. Independent variables: family support and social connectivity

This study measured family support as structural, instrumental, and emotional support. Structural family support was represented by living arrangements. Living arrangement was measured by whether the participants lived with any of their children (0 means no and 1 means yes). Instrumental support from children was measured by one question: “Are your children able to accompany you to see the doctor?” (0 means no, 1 means yes). Perception of children’s filial piety was measured by one question: “In general do you think that your children are filial?” Response options ranged from 1 (not at all) to 5 (very much). Due to the skewed distribution of this variable, it was dummy coded as 1 means filial and 0 means not filial in the final analysis.

The social connectivity was measured using Lubben Social Network Scale-6 (LSNS-6) (41). It is divided into two dimensions: relatives and friends. The LSNS-6 includes 6 items that measure the size of active and intimate networks of family and friends with whom they could talk or call for help. The LSNS-6 is constructed from a set of three questions that assess kinship ties and a comparable set of three questions that assess friend ties. The items related to kinship include 3 questions: (1) How many relatives do you see or hear from at least once a month? (2) How many relatives do you feel close to such an extent that you could call on them for help? (3) How many relatives do you feel at ease with that you can talk to about private matters? Those three questions are repeated with respect to non-kin ties by replacing the word relatives with the word friends. The maximum score of the LSNS-6 is 30, which is an equally weighted sum of the six items. An LSNS-6 family subscale is constructed from the three LSNS-6 questions on relatives. Similarly, an LSNS-6 friends subscale is constructed from the three questions on friends.

#### 2.2.3. Covariates

Sociodemographic variables included age, sex, education level, marital status, sense of economic security, self-rated health, alcohol drinking, smoking, and subjective age. Age was measured in years and included as a covariate as a continuous variable. Sex contains both males and females. Educational level was classified as illiteracy, junior high school and below, and high school and above. Marital status was recoded as not being married and being married. Sense of economic security was measured by asking “Do you feel you are currently financially secure?” with options including “no” and “yes.” Self-rated health is a comprehensive evaluation index for the elderly’s own physical health, body function, and psychosocial function. Self-rated health was measured by asking “how do you feel about your own health now?.” Options include: “very good,” “good,” “fair,” “bad,” and “very bad,” and were assigned values of “5,” “4,” “3,” “2,” “and 1” respectively. Self-rated health was used as a continuous in our analysis. Smoking and alcohol drinking status was recoded as current and former/never user. Smoking status was measured by asking “Do you have the habit of smoking?” Combine options like “Never,” “Once, do not smoke now,” “Occasionally,” “Frequently,” etc. into “Current” and “Before/Never.” Drinking status was measured by asking “Do you have a drinking habit?” Combine options like “Never,” “Once, but not now,” “Occasionally,” and “Frequently” into “Current” and “Before/Never.” Subjective aging was measured by asking “Do you feel old now?” with options including “old” and “not old.”

### 2.3. Statistical analysis

Participants’ descriptive characteristics were assessed using means and standard deviations for continuous variables and numbers and percentages for categorical variables. Descriptive analysis was conducted by urban–rural differences using chi-square tests and independent *t*-tests. Multiple linear regressions were conducted to examine the associations between different types of family support and social connectivity and depressive symptoms, controlling for sociodemographic covariates. The interaction effects of urban–rural status with five variables (live with family members, have instrumental support from children, perception of children’s filial piety, social connectivity with family, social connectivity with friends) were also examined in regression models. Data were analyzed using R version 4.2.1. A significance level of 0.05 was employed.

## 3. Results

### 3.1. General characteristics of the sample

Table 1 describes the general characteristics of the sample. The mean age of the sample was 72.98 (SD: 7.57) years. 50.8% of the sample was male and 49.2% of the sample was female. The education level of most participants was junior high school and below. Most participants (65.0%) were married. For the sense of economic security, 70.4% of the sample felt they are currently financially secure. For self-rated health, most participants reported fair (51.2%) or good (31.0%) health. For subjective aging status, most participants (76.2%) reported they feel they are old now. For lifestyle habits, 31.8% of participants drank alcohol, and 25.5% smoked. For family support, 82.3% of participants reported they were living with their family members; 90.3% of participants reported they received instrumental support from their children; 77.5% of participants reported they think that their children exhibited filial piety. With respect to social connectivity, the score for social connectivity with family was 12.40 (SD, 8.99), and social connectivity with friends was 6.70 (SD, 8.43). With respect to urban–rural differences, there were differences in educational level, sense of economic security, smoking status, subjective aging, living with family members, perception of children’s filial piety, social connectivity with friends. Older adults in urban areas were more educated than those in rural areas (*p* < 0.001). Older adults in urban areas reported higher levels of economic security than those in rural areas (*p* < 0.001). Older adults in rural areas were more likely to smoke than those in urban areas (*p* < 0.01). Older people in urban areas had a higher degree of subjective aging than those in rural areas (*p* < 0.001). For family support and social connectivity, older adults in urban areas were more likely to live with family members (*p* < 0.001), think their children exhibited filial piety (*p* < 0.05), and had more social connectivity with friends (*p* < 0.05) than those in rural areas (See Table 1).

**Table 1:** Descriptive characteristics of the sample (*N* = 824).

Variables	Categories	Mean (SD)/*n* (%)	Rural (*n* = 421)	Urban (*n* = 403)	*p*-value
Age (years)		72.98 (7.57)	73.01 (7.55)	72.94 (7.61)	0.889
Sex	Male	419 (50.8)	228 (54.2)	191 (47.4)	0.061
	Female	405 (49.2)	193 (45.8)	212 (52.6)	
Education level	Illiteracy	302 (36.7)	224 (53.2)	78 (19.4)	<0.001
	Junior high school and below	451 (54.7)	196 (46.6)	255 (63.3)	
	High school and above	71 (8.6)	1 (0.2)	70 (17.4)	
Marital status	Not married	288 (35.0)	160 (38.0)	128 (31.8)	0.071
	Married	536 (65.0)	261 (62.0)	275 (68.2)	
Sense of economic security	No	244 (29.6)	180 (42.8)	64 (15.9)	<0.001
	Yes	580 (70.4)	241 (57.2)	339 (84.1)	
Self-rated health	Very poor	29 (3.5)	18 (4.3)	11 (2.7)	0.079
	Poor	117 (14.2)	71 (16.9)	46 (11.4)	
	Fair	422 (51.2)	203 (48.2)	219 (54.3)	
	Good	222 (26.9)	109 (25.9)	113 (28.0)	
	Very good	34 (4.1)	20 (4.8)	14 (3.5)	
Alcohol drinking	No	562 (68.2)	282 (67.0)	280 (69.5)	0.488
	Yes	262 (31.8)	139 (33.0)	123 (30.5)	
Smoking status	No	614 (74.5)	292 (69.4)	322 (79.9)	<0.01
	Yes	210 (25.5)	129 (30.6)	81 (20.1)	
Subjective aging	Old	628 (76.2)	77 (18.3)	119 (29.5)	<0.001
	Not old	196 (23.8)	344 (81.7)	284 (70.5)	
Live with family members	No	146 (17.7)	98 (23.0)	48 (11.9)	<0.001
	Yes	678 (82.3)	323 (76.7)	355 (88.1)	
Instrumental support from children	No	80 (9.7)	44 (10.5)	36 (8.9)	0.536
	Yes	744 (90.3)	377 (89.5)	367 (91.1)	
Perception of children’s filial piety	No	185 (22.5)	108 (25.7)	77 (19.1)	<0.05
	Yes	639 (77.5)	313 (74.3)	326 (80.9)	
Social connectivity with family		12.40 (8.99)	12.05 (7.80)	12.77 (10.08)	0.251
Social connectivity with friends		6.70 (8.43)	6.00 (7.49)	7.43 (9.27)	<0.05

### 3.2. The regression analysis on depression symptoms by urban–rural subgroups

In rural areas, respondents who felt their children exhibited filial piety were more likely to report fewer depression symptoms (*β* = −1.512, *p* < 0.001). Respondents who had more social connectivity with family were more likely to report fewer depression symptoms (*β* = −0.074, *p* < 0.001). For sociodemographic variables, respondents who were younger (*β* = 0.044, *p* < 0.05), whose education level was junior high school and below (*β* = −0.713, *p* < 0.05), had a sense of economic security (*β* = −1.594, *p* < 0.001), had better health status (*β* = −2.977, *p* < 0.001, very good self-rated health) were more likely to report fewer depression symptoms (Table 2).

**Table 2:** Results of multiple linear regression analysis on depression symptoms in rural and urban areas.

Variables	Categories	Rural		Urban	
		*β* (SE)	*p*-value	*β* (SE)	*p*-value
Age (years)		0.044 (0.021)	<0.05	0.002 (0.018)	0.898
Sex	Male	Ref		Ref	
	Female	0.339 (0.316)	0.285	0.301 (0.294)	0.307
Education level	Illiteracy	Ref		Ref	
	Junior high school and below	−0.713 (0.296)	<0.05	0.049 (0.335)	0.884
	High school and above	−1.038 (2.573)	0.687	−0.504 (0.453)	0.267
Marital status	Not married	Ref		Ref	
	Married	−0.262 (0.348)	0.452	−0.401 (0.324)	0.217
Sense of economic security	No	Ref		Ref	
	Yes	−1.594 (0.265)	<0.001	−2.787 (0.329)	<0.001
Self-rated health	Very poor	Ref		Ref	
	Poor	0.417 (0.678)	0.538	−2.494 (0.792)	<0.01
	Fair	−1.800 (0.644)	<0.01	−5.501 (0.731)	<0.001
	Good	−2.075 (0.690)	<0.01	−6.365 (0.760)	<0.001
	Very good	−2.977 (0.877)	<0.001	−7.451 (0.962)	<0.001
Alcohol drinking	No	Ref		Ref	
	Yes	−0.376 (0.339)	0.267	0.386 (0.282)	0.171
Smoking status	No	Ref		Ref	
	Yes	0.039 (0.333)	0.908	−0.606 (0.327)	0.065
Subjective age	Old	Ref		Ref	
	Not old	0.672 (0.350)	0.055	0.875 (0.280)	<0.01
Live with family members	No	Ref		Ref	
	Yes	0.340 (0.355)	0.338	0.409 (0.417)	0.327
Instrumental support from children	No	Ref		Ref	
	Yes	−0.267 (0.424)	0.529	−1.276 (0.442)	<0.01
Perception of children’s filial piety	No	Ref		Ref	
	Yes	−1.512 (0.309)	<0.001	−0.836 (0.321)	<0.01
Social connectivity with family		−0.074 (0.019)	<0.001	−0.012 (0.013)	0.347
Social connectivity with friends		0.011 (0.020)	0.571	−0.040 (0.014)	<0.01

In urban areas, respondents who received instrumental support from their children were more likely to report fewer depression symptoms (*β* = −1.276, *p* < 0.01). Respondents who thought their children exhibited filial piety were more likely to report fewer depression symptoms (*β* = −0.836, *p* < 0.01). Respondents who had more social connectivity with friends were more likely to report fewer depression symptoms (*β* = −0.040, *p* < 0.01). For sociodemographic variables, respondents who had a sense of economic security (*β* = −2.787, *p* < 0.001), had better health status (*β* = −7.451, *p* < 0.001, very good self-rated health), felt old (*β* = 0.875, *p* < 0.01) were more likely to report fewer depression symptoms (Table 2).

### 3.3. The relationship between family support and social connectivity and depression symptoms by an urban–rural difference

In the fully adjusted regression model, the more social connectivity with family was associated with decreased depressive symptoms (*β* = −0.074, *p* < 0.001), although interaction analyses revealed this association to be mitigated among urban-dwelling participants (*β* = 0.053, *p* < 0.05). While social connectivity with friends was similarly associated with decreased depressive symptoms (*β* = −0.040, *p* < 0.01), in this case, urban-dwelling participants reported a greater reduction of depressive symptoms (*β* = −0.053, *p* < 0.05) (Table 3).

**Table 3:** Regression of main effect and interactions for depression symptoms.

Variables	*β* (SE)	*p*-value
Residence		
Rural	Ref	
Urban	0.396 (0.744)	0.595
Live with family members		
No	Ref	
Yes	0.297 (0.270)	0.271
Instrumental support from children		
No	Ref	
Yes	−0.149 (0.408)	0.714
Perception of children’s filial piety		
No	Ref	
Yes	−1.312 (0.295)	<0.001
Social connectivity with family	−0.067 (0.018)	<0.001
Social connectivity with friends	0.014 (0.019)	0.447
Urban Í live with family members	0.160 (0.478)	0.737
Urban Í having instrumental support from children	−1.141 (0.616)	0.064
Urban Í children being filial	0.365 (0.447)	0.414
Urban Í social connectivity with family	0.053 (0.023)	<0.05
Urban Í social connectivity with friends	−0.055 (0.023)	<0.05

Adjusted for age, sex, educational level, marital status, living alone or not, sense of economic security, self-rated health, alcohol drinking, smoking status, and subjective age.

## 4. Discussion

Overall, the results of this study suggested that older adults both in rural and urban areas with strong social connectivity (both with friends and family) displayed fewer depressive symptoms. Importantly, it was revealed that living in an urban area weakened the effect of social connectivity with family on reducing depressive symptoms, while strengthening the effect of social connectivity with friends on reducing depressive symptoms. These findings align with the initial hypothesis and emphasize the importance of tailoring social interventions within urban and rural China to the unique types of social dynamics occurring in both settings.

Findings suggested that in rural areas, respondents who think their children exhibited greater filial piety were more likely to report fewer depression symptoms, consistent with previous studies suggested a significant and negative relationship between a child’s filial piety levels and older parents’ depressive symptoms after controlling for sociodemographic variables (42, 43). This finding supports the notion that filial piety, as a traditional Asian social and cultural norm, still plays an important role in healthy aging in China (42, 44). Studies in Asia, America, Europe and other countries have also found that respondents with greater family connectivity were more likely to report fewer depression symptoms (45–48), which was also observed in our study (particularly among Chinese older adults living in rural areas). In urban areas, respondents who received instrumental support from their children were more likely to report fewer depression symptoms, similar to past research which has suggested an inverse relationship between receiving instrumental support from children and parental depression (49, 50). Respondents who thought their children exhibited greater filial piety were more likely to report fewer depression symptoms (also observed among rural-dwelling parents). Indeed, a systematic literature review and other studies (51–53) also shown filial piety to be negatively associated with depression in older people. Moreover, findings that greater connectivity with friends was associated with fewer depression symptoms may be explained by past research suggesting higher level of contact with friends to be associated with increased emotional support from those friends (54). Indeed, one study also observed that subjective social isolation from friends was associated with more depressive symptoms (13). For sociodemographic variables, respondents who had a sense of economic security, had better health status were more likely to report fewer depression symptoms both among urban and rural elderly. Research indicates that economic insecurity had a stronger association with somatic symptoms. These symptoms have been shown to adversely impact a person’s mental well-being and can lead to mental disorders (like anxiety and depression), which was also verified in this study (55).

Although it was hypothesized that less multigenerational living and interactions would occur between older adults and family members in urban settings, it was observed that older adults in urban areas were more likely to live with family members, think their children exhibited filial piety (albeit still having more social connectivity with friends) than those in rural areas. This high connectivity with family members was observed in urban settings despite familial connectivity having a lesser effect on reducing depression compared to rural settings, suggesting that the types of family interactions in such settings (rather than simply their overall degree) may be important to consider in the context of health promotion, which was also investigated in an study about the impact of specific shared family activities on the health of young Asian Americans (56). Moreover, one study using data from the China Health and Longitudinal Study (CHARLS) examined the moderating effect of rural or urban residence on the association between social support and depressive symptoms among Chinese adults and found that across all types of social support, the degree of association with changes in depressive symptoms was not significantly different between urban and rural areas (57), which differed from our study findings. This difference may be explained by the types of psycho-social scales used; we used the GDS-15 and while the prior used the Chinese version of a 10-item center for epidemiologic studies depression scale. Additionally, the prior study did not make systematically distinguish between social support from friends and family (as done in our study). Greenfield (58) developed a theory of social change and human development in which social changes alter cultural values and learning environments, thereby altering developmental paths. Social change and human development theory predicts that there will be a greater emphasis on individualistic values and independence when people live in urban areas, and a greater emphasis on collectivistic values and interdependence when people live in rural areas. As Kendall says, modernization is related to the process of urbanization and industrialization and the spread of education (59). In critical theory of sociology, modernization is a process of overall rationalization. As the degree of modernization within society increases, the individual becomes more and more important, eventually replacing the family or community as the basic unit of society. In addition, migration under modernization theory has some impacts and challenges on traditional culture. Western culture and traditional Chinese Confucian culture have had some blending, making this difference between urban and rural areas appear. As far as the social security system is concerned, due to the dual structure of urban and rural areas, there are many inequalities between urban and rural areas, such as health inequality. Moreover, residents in urban and rural areas have some differences in access to health services and available social welfare systems, which may strengthen the differences in the ways of obtaining social support between urban and rural areas. Therefore, we should expect that urban Chinese older adults value independence more than rural older adults, and are more likely to seek support from friends to relieve depression. In contrast, rural Chinese older adults valued interdependence more than urban older adults and were more likely to seek support from family relatives to relieve depression.

This study has some limitations. First, the variables included in this study cannot fully explain all aspects of social support among older adults. For example, the study did not include financial support from children or support from spouses and the typical types of family interactions. Second, causal relationships between family support and social connectivity, and depressive symptoms cannot be inferred because this is a cross-sectional study. Future research should consider a prospective longitudinal design with a more comprehensive array of relevant variables. Finally, the study sample size was relatively small and only representative of the Jiangsu situation, however, the rigorous PPS sampling method to enhance the representativeness of the data is a strength of the analysis.

## 5. Conclusion

Our findings provide preliminary support for the important social and health disparities experienced across urban–rural lines in China, as well as the relationship between family support and social connectivity and depression among Chinese older adults. It was revealed that for the urban-dwelling Chinese older adults, social interventions involving connectivity with friends may be more effective in alleviating depressive symptoms, while for the rural-dwelling older adults, interventions focused on family connectivity may be particularly effective. Although both urban and rural populations in China have undergone rapid modernization and social change, they have experienced different degrees of modernization and cultural change, thus large differences the social environment and cultural norms may remain between urban and rural areas (60). As China continues to urbanize, more attention may be needed on growing disparities between urban and rural regions, and the need for tailored interventions in both types of regions that consider the unique factors that affecting the physical and mental health of older adults. As a next step, further qualitative and mixed-methods research is also needed to disentangle the specific mechanisms which may explain the differing associations observed in the study, and the social norms and behaviors underlying the protective effect of social connectivity against depression.

## Data availability statement

The raw data supporting the conclusions of this article will be made available by the authors, without undue reservation.

## Ethics statement

The studies involving human participants were reviewed and approved by the China Research Center on Aging. The patients/participants provided their written informed consent to participate in this study.

## Author contributions

YG and AG contributed to the study’s conception and design. Material preparation and analysis were performed by YG, SA, and AG. The first draft of the manuscript was written by YG. YG, SA, and AG commented on previous versions of the article. All authors contributed to the article and approved the submitted version.

## Funding

This study was funded by the Major Project of Philosophy and Social Science Research in Colleges and Universities of Jiangsu Province (grant number: 2022SJYB0236).

## Conflict of interest

The authors declare that the research was conducted in the absence of any commercial or financial relationships that could be construed as a potential conflict of interest.

## Publisher’s note

All claims expressed in this article are solely those of the authors and do not necessarily represent those of their affiliated organizations, or those of the publisher, the editors and the reviewers. Any product that may be evaluated in this article, or claim that may be made by its manufacturer, is not guaranteed or endorsed by the publisher.
